# Splenic abscess owing to cancer at the splenic flexure: A case report and comprehensive review

**DOI:** 10.1097/MD.0000000000004941

**Published:** 2016-09-23

**Authors:** Gavish K. Awotar, Fuwen Luo, Zhengdong Zhao, Guoxin Guan, Shili Ning, Jinshuai Ren, Yaqing Liu, Guangzhi Wang, Pixu Liu

**Affiliations:** a Department of General Surgery, The Second Affiliated Hospital of Dalian Medical University, Dalian City, Liaoning Province, 116023, P.R. China; b The First Affiliated Hospital Collaborative Innovation Center of Oncology-Institute of Cancer Stem Cell, Dalian Medical University, Dalian City, Liaoning Province, P.R. China.

**Keywords:** colorectal cancer, percutaneous image-guided drainage, splenectomy, splenic abscess, splenic flexure, splenocolic fistula

## Abstract

**Background:**

The cancer of the splenic flexure of the colon is a rare medical entity with severe morbidity because of its insidious onset.

**Methods:**

We present the case of a 59-year-old male patient with dull left upper quadrant pain, leukocytosis, and anemia. A splenic abscess described as an air-fluid level with splenocolic fistula was found on CT scan imaging. Surgery was done for splenic pus drainage. He was again admitted 2 months later for intestinal obstruction.

**Results:**

An exploratory laparotomy showed multiple hard, gray liver nodules as well as a hard mass in the small bowel. Owing to extensive adhesions and a late stage of cancer involvement, the splenic flexure tumor was not resected. A loop transverse colostomy was done and a Coloplast^TM^ Colostomy bag placed. We also reviewed the literature-linking colon cancer and splenic abscess with specific attention to the carcinoma of the splenic flexure. As the latter invades through the spleen matter, there is the creation of a splenocolic fistula, which allows the migration of normal gut flora into the spleen. This leads to the formation of the splenic abscess.

**Conclusion:**

This is the 13^th^ case report pertaining to invading colonic cancer causing a splenic abscess. Although the treatment for splenic abscesses is shifting from splenectomy to image-guided percutaneous pus drainage, the few reported cases make the proper management of such complication still unclear.

## 1 Introduction

The spleen is one of the usually forgotten organs to be considered in the work-up of a differential diagnosis during clinical evaluation. Splenic abscess can be seen as that rare entity that has or hides disastrous underlying pathology. With autopsy studies revealing 0.14% to 0.7% of splenic abscess,^[1–3]^ the most commonly affected population are males and immunosuppressed patients,^[3–6]^ with the most common cause being typhoid fever in the preantibiotic era and nowadays AIDS, abdominal infections, pneumonia, bacterial endocarditis, and urogenital infections.^[7]^ The rise in parasitic abscesses and fungal abscesses is because of increased international travel, whereas increase in immunosuppression is because of the increasing number of AIDS patients, as well as subjects undergoing organ transplant or affected by neoplastic diseases.^[8,9]^ Direct invasion of the spleen by colon cancer of the splenic flexure of the colon is not a common occurrence. We report the case of a cancer of the splenic flexure of the colon, which created a splenocolic fistula to cause an abscess in the spleen. To have an initial presentation of colon cancer as splenic abscess is not common in daily practice and young surgeons should again be remembered to consider all surrounding organs while consulting a complaint, especially in the abdomen. After the case report, the various literature reports of cases of similar nature are exposed for a better understanding for this uncommon but yet, morbid complication of colon cancer.

## 2 Presenting concerns

We present the unfortunate case of a 59-year-old Chinese man (Dalian, China) with no relevant medical history. He initially presented to our department in the beginning of March 2015, complaining of intermittent dull left upper quadrant pain, exacerbated with exertion, with no radiation and intermittent low-grade fever not exceeding 37.6°C over the last month, but which have aggravated over the 2 days before admission. During the 1 month, he sought help in primary-level health care facilities, but he was given only anti-inflammatory drugs and sent home, without further investigations.

## 3 Clinical findings

Upon initial examination, his vitals were stable except a low-grade fever. Cachexic in nature, his physical examination was also significant for tenderness in the left upper quadrant region with no organomegaly felt.

## 4 Timeline

**Figure 1:**
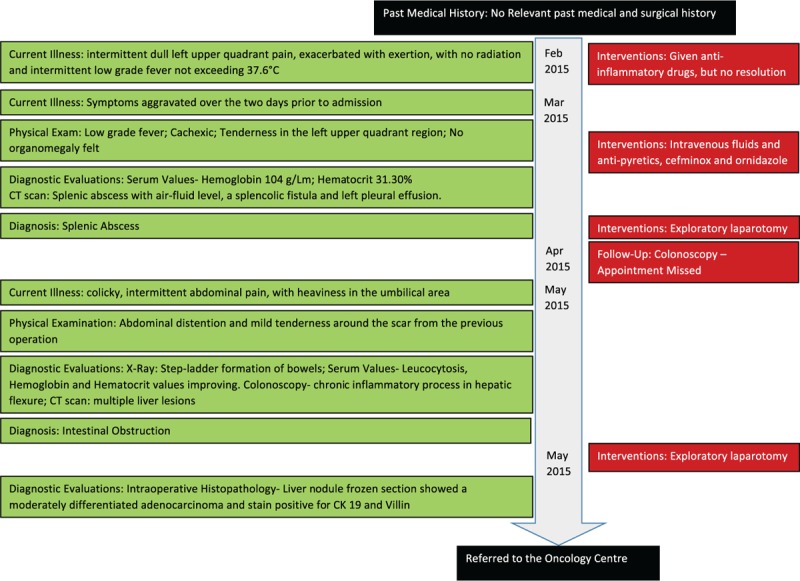
Timeline of patient's course from admission to surgery.

The timeline of events is as shown in Figure 1.

## 5 Diagnostic focus and assessment

**Figure 2:**
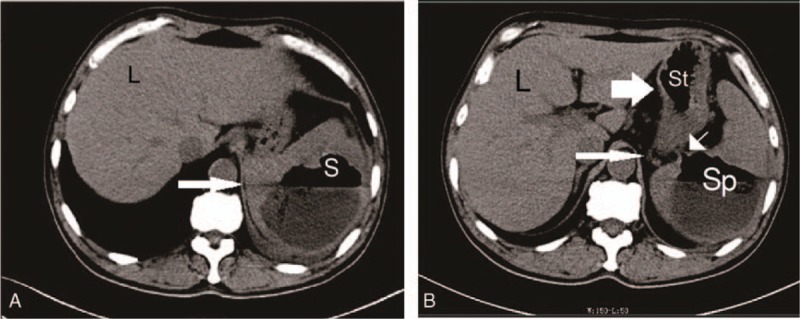
(A) In the CT Scan upon first admission, the white arrow shows the distinct air-fluid level seen within the spleen. L: Liver; S: Spleen. (B) Splenocolic fistula (small arrow) and its relation with the colon (thin white arrow) and stomach (thick white arrow). L: Liver; Sp: Spleen; St: Stomach.

He had leukocytosis with a left shift, hemoglobin level of 104 g/L and hematocrit (HCT) of 31.30%. Computed tomography (CT) scan (Figure 2) revealed a splenic abscess with air-fluid level, a splencolic fistula, and left pleural effusion. We believed that the primary infection was in the spleen, which then has caused an inflammatory process involving the splenic flexure of the colon, thus leading to the splenocolic fistula. The patient was given adequate amounts of intravenous fluids and started on a course of 2 g of cefminox infusion thrice daily and ornidazole 1 mg daily for 7 days.

## 6 Therapeutic focus and assessment

An exploratory laparotomy with a 40-cm left anterolateral incision revealed a 5-cm hard immovable mass in the region of the splenic flexure of the colon with a spleen of size 12 cm. The whole compound was surrounded by extensive adhesions to the stomach, tail of pancreas, and diaphragm. Three hundred microliters of pus was drained intraoperatively from the spleen with copious amount of necrotic debris from the spleen and the splenocolic fistula was closed. Examination of the liver, gall bladder, stomach, mesentery, and abdominal aorta was not significant, with no lymph nodes nor nodules felt.

As the surgeon decided that a splenectomy would not have been beneficial to the patient already cachexic in nature, the spleen was not removed. However, a colonoscopy was ordered to be done after the patient recovers from the surgery. Three drainage tubes were placed in situ before standard technique layer-by-layer closure of the abdomen. The patient recovered well postoperatively with no complications up to his discharge from the hospital. One drainage tube was removed after 1 week as there was no drainage and the others were kept until the follow-up visit at the end of April, as they were still draining.

**Figure 3:**
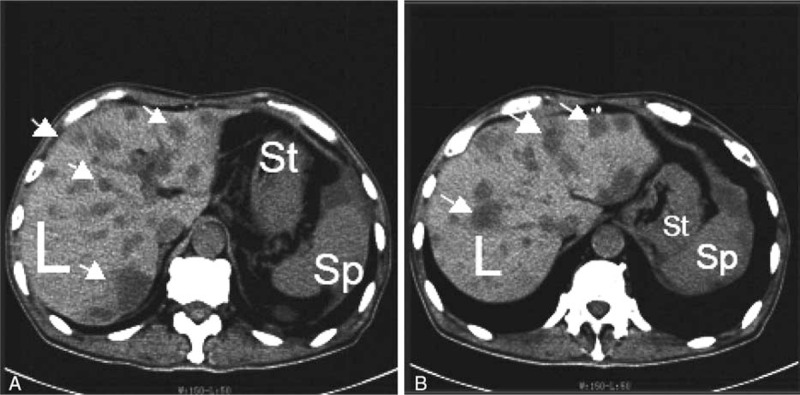
CT Scan of patient on second admission. (A & B) Presence of multiple liver lesions (arrows). L: Liver; Sp: Spleen; St: Stomach.

Unfortunately, the patient did not attend his follow-up visit and the colonoscopy was not performed. However, 2 months later, he came again to the department complaining of 1-day history of colicky, intermittent abdominal pain, with heaviness in the umbilical area, having vomited 3 times undigested food and mucous, and not passing stools for 1 day, with no improvement after the use of self-administered laxatives. He has no fever and no other complaints. An anterior-posterior erect abdominal x-ray in the Emergency Department showed step-ladder formation of the bowels with the presence of 4 air-fluid level bubbles. He was primarily admitted for incomplete intestinal obstruction. His vitals were stable and abdominal examination did not reveal any abnormal finding except for abdominal distention and mild tenderness around the scar from the previous operation. Upon admission, his blood parameters showed a leukocytosis with a left shift, whereas his hemoglobin and hematocrit values were improving. A colonoscopy was ordered and the relevant findings were chronic inflammation in the region of the hepatic flexure, with nothing noted at the splenic flexure. Biopsies of the relevant region were sent for pathological studies, which later confirmed a chronic inflammatory process. CT scan (Fig. 3) showed multiple liver lesions that we thought to be liver abscess from the unresolved splenic abscess. He was put on conservative treatment for intestinal obstruction as per hospital protocol, which included insertion of a nasogastric tube, nil by mouth, total parenteral nutrition, and 4-hourly monitoring of vitals. He was also given cefminox for the liver abscess.

Forty-eight hours later, the condition of the patient did not improve and his cancer serum markers results showed elevated values for CEA 49.66 μg/L (normal: 0.00–5.00 μg/L), CA 125 117.90 KU/L (normal 0.00–35.00), CA 199 265.94 KU/L (normal 0.00–35.00), and CA 242 65.54 (normal 0.00–20.00).

Surgery was chosen. A 20-cm upper median incision was done for the exploratory laparotomy. Within the peritoneal cavity, around 500 mL of yellow fluid was drained with a sample sent to the laboratory. The bowels were dilated with thick edematous walls with the external diameter of the dilated small intestines being around 4 cm. The greater omentum was adherent to the left side of the peritoneal cavity, covering the transverse colon and part of the small intestines. Within the mesentery of the small intestine and greater omentum, multiple hard nodules were felt. Ten centimeters after the Treitz's ligament, the small intestine had a hard consistency. The liver was felt and hard grayish nodules were found, with the largest being 3 cm in the inferior border of the liver, with no abnormality found in the stomach, duodenum, spleen, kidney, gall bladder as well as no metastatic involvement of the peri-aortic lymph nodes. However, the pelvic floor had metastatic seedlings. The intraoperative diagnosis was sT4NxM1. Adhesiolysis between the small intestines was performed and the small intestines were decompressed with a lot of gas and yellow stools being evacuated at an incision 20 cm before the ileocecal valve, which was sutured closed hereafter. The invaded small bowel was not resected and a side-to-side intestinal tension-free anastomosis with the jejunum was done. A right partial hepatectomy with nodule resection was performed and frozen section sent for pathology, which revealed a moderately differentiated adenocarcinoma and stain positive for CK 19 and Villin. Owing to extensive adhesions and a late stage of cancer involvement, the tumor felt at the splenic flexure of the colon was not resected. A loop transverse colostomy was done and a Coloplast^TM^ Colostomy bag placed.

## 7 Follow-up and outcome

Postoperatively, the patient recovered without complications with timely dressing. With the sad occurrence of liver metastatic, there is nothing more that surgery can do and he was then referred to the Oncology Center.

## 8 Discussion

### 8.1 The splenic flexure

As compared in Table 1,^[10]^ Bacon et al^[11]^ in 1952 and Popper^[12]^ in 1968 reported the incidence of carcinoma of the splenic flexure to account for 2% to 3% of all colorectal cancer. In 143,747 patients studied between 1995 and 2009,^[10]^ the incidence of carcinoma of the splenic flexure ranged between 2.80% and 3.11% with respect to age groups and sex. Taking into consideration the time intervals mentioned in the studies,^[10–12]^ the incidence of colon cancer of the splenic flexure has remained stable in the region of 2% to 3% of total colon cancers.

**Table 1:**
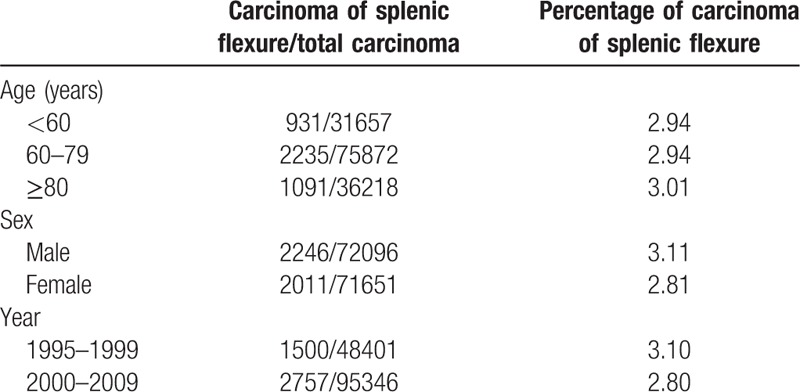
Incidence of the carcinoma of the splenic flexure with respect to age and sex^[12]^.

Colorectal cancer had a prevalence of 16.3% in males and 12.2% in females, being the fifth cancer with the highest incidence in both sex groups.^[13]^ However, specific data about the involvement of the splenic flexure in malignancy were not available.

The splenic flexure is the part of the colon that is most inaccessible to proper exposure during operations. It lacks a posterior peritoneal investment where it lies on the perirenal fat and fascia on the outer border of the left kidney.^[14]^ It is attached to the tail of the pancreas by the left end of the transverse mesocolon. Above and to the left, the lower border of the spleen is supported by the phrenocolic ligament, which connects the splenic flexure to the diaphragm. It is supplied by the left branches of the midcolic vessels and the upper branches of the left colic vessels. Lymphatic drainage of the splenic flexure is through to the hilum of the spleen and along the tail of the pancreas.^[15]^ Based on this, the appropriate treatment of the carcinoma of the splenic flexure should include splenectomy with distal pancreatectomy.^[16]^

### 8.2 Splenic abscess

#### 8.2.1 Risk factors

The mortality rate of splenic abscesses ranges between 13% and 25%.^[3,17–19]^ Direct invasion from neighboring neoplasms to the spleen is a rare entity. In our patient, *Enterobacter cloacae* was the organism cultured from the pus that was drained during the surgical intervention. The pus formation was because of to the direct invasion of the gut flora through the splenocolic fistula. There are 5 distinct predisposing factors in descending order^[20]^: metastatic infections; trauma; contiguous infections; hematological disorders; immunodeficiency states (including AIDS).

Metastatic infection accounts for approximately two-thirds of all splenic abscesses reported in the literature, with endocarditis and secondary bacterial seeding of the spleen constituting the most common 2 etiologies.^[20]^ The most common organisms involved in the formation of splenic abscesses, in most series, were aerobic microbes, especially staphylococci, streptococci, *Salmonella*, and *Escherichia coli*.^[3,21,22]^ Furthermore, the cultures of organisms can reflect the pathogenesis of the original causative insult.^[23]^*S aureus* and *S bovis* were associated with endocarditis, *K pneumoniae* with respiratory infection or liver abscess, *E coli* with urinary tract and abdominal infection, and *Bacteroides* spp and *Clostridium* spp with abdominal infection.^[21,23]^ While considering the microorganism of the initial insult that can lead to splenic abscess, one should also remember the geographical distribution of the causative agents. In one of the largest series of splenic abscess reported in the literature by Chang et al^[6]^ in 2006 in Taiwan, analyzing 67 cases over 19 years, *K pneumoniae* was the most common pathogen, which was similar to other Asian countries. Splenic trauma causes a physical breach that increases risk of infection in the spleen leading to splenic abscess. An interrupted splenic capsule may complicate the catheterization during ultrasound-guided percutaneous drainage.^[20]^ In the English literature for splenic abscess owing to *M tuberculosis*, older American studies reported at most 0.8% of incidence compared to 1.5% and 7.8% in the more modern Southern Asian reports.^[22]^ This may be because of the increased prevalence of the disease caused by *M tuberculosis* in those countries.^[22]^ Fungal infections causing splenic abscess are also on the rise, with *Candida* being the most common, especially in the immunocompromised patients.^[22]^ Hemoglobinopathies, especially sickle cell disease, leukemia, polycythemia, or vasculitis, can cause splenic infarction, which can be infected and evolve into splenic abscesses.^[20]^ Owing to the increase in the number of AIDS and organ transplant patients,^[6,24]^ the advent of splenic abscess because of immunosuppression has increased; 33.5% of patients in a review involving 287 patients^[3]^ had an immunosuppressed state with nearly half of the 287 patients being intravenous drug abusers and afflicted by AIDS.

#### 8.2.2 Clinical presentation

Our patient complained of intermittent dull left upper quadrant pain, exacerbated with exertion, with no radiation and intermittent low-grade fever not exceeding 37.6°C over the last month, but these symptoms have aggravated over the 2 days before admission.

The clinical presentation of splenic abscess is always vague^[20,22,25]^ and the triad of fever, left upper quadrant pain, and a tender mass for the diagnosis of splenic abscess by Sarr and Zuidema^[26]^ were not present in our patient. This nonspecificity of symptoms unfortunately prolongs the delay in diagnosis,^[22,25]^ with the average time between the onset of symptoms and the diagnosis exceeding 2 weeks.^[7,27]^

Splenic abscess should always be suspected in a patient with pyrexia of unknown origin (PUO), which is the main symptom in 90% of cases in previous reported series.^[2,24,28,29]^ Left hypochondriac pain and/or tenderness was/were not reliable signs because they were present in 50% to 70% of cases only, and splenomegaly present in 30% only.^[3,7,29]^ Atypical signs such as digestive problems, vomiting or left-sided pulmonary symptoms were rare in occurrence.^[3,7,9,28]^

Leukocytosis was noted in 60% to 100% of cases.^[3,29,30]^ On both admissions, our patient had raised leukocytosis with increased neutrophil count. This is in accordance with the infective process within the spleen over the month that he has harbored the disease.

Roentogram of the chest may show left pleural effusion, raised left hemi-diaphragm or atelectasis of the left lung lower lobe.^[3]^

The definitive diagnosis is through the use of imaging modalities.^[3,31]^ The combination of ultrasound with CT scan approximates the success of diagnosis to nearly 100%.^[24,29,32]^ Our patient presented with low-grade fever and left upper quadrant pain only while no mass was felt. However, the diagnosis was made with the help of CT scan imaging, which revealed the unifocal splenic abscess with the presence of an air-fluid level and left pleural effusion upon his first admission.

Infectious and inflammatory diseases form the major part of cases with multifocal splenic lesions.^[33]^ Bacterial lesions can be solitary, multiple, or multiloculated. Multiple lesions are usually centrally located, circular, or irregular in shape with low attenuation centers of fluid/necrosis.^[33]^ When a capsule has developed, there can be minimal peripheral enhancement. The presence of gas is diagnostic, even though it is rarely found.^[34]^ In our case, the air fluid level was present together with the splenocolic fistula, thus establishing a straight-forward diagnosis. However, the interpretation of imaging studies of the spleen remains tricky and Table 2 provides a brief overview of the different defining features of the spleen under different imaging modalities.^[33]^

**Table 2:**
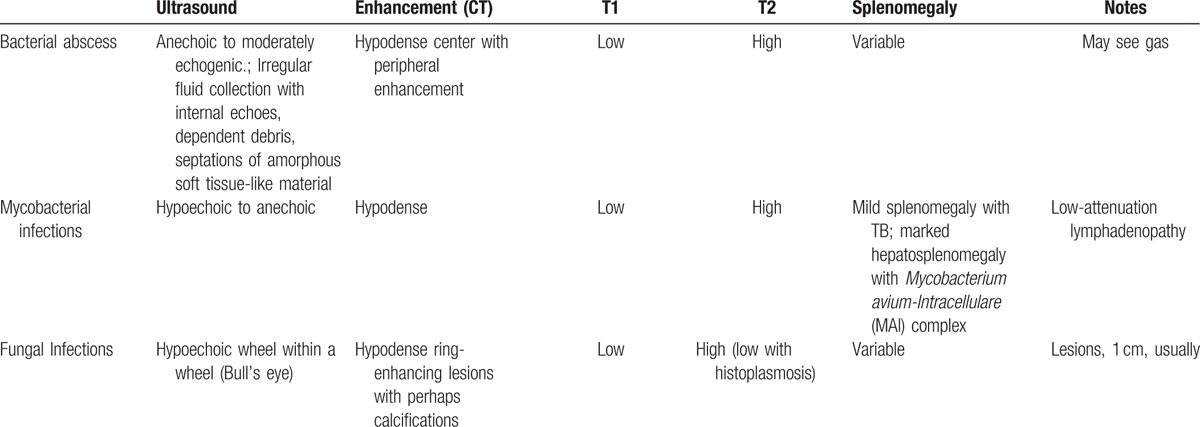
Differential diagnosis of splenic lesions on imaging studies^[33]^.

Blood cultures are mandatory in patients in PUO. In cases of splenic abscess, blood cultures are positive in 48.2% to 71.8% of cases,^[3,6]^ but can drop to below 30% when the population consists of patients with tuberculosis and AIDS.^[22,27,35]^

Together with blood cultures, which are usually monomicrobial in nature, a fine needle aspiration of the pus from the splenic abscess for culture is essential. Diagnostic fine needle aspiration is important to identify the proper course of antibiotics to follow. Usually, this sample is polymicrobial in almost half of cases.^[4]^ In the immunocompromised, fine needle aspiration may also help in differentiating splenic abscess from other splenic lesions that have a similar radiographic interpretation, such as lymphoma, metastasis, infarction, or hematoma.^[4]^ Unfortunately, this was not performed in our patient. The pus was collected intraoperatively and thereafter revealed a monomocrobial culture of *Enterobacter Cloacae*.

#### 8.2.3 Treatment

Antibiotics and splenectomy were traditionally considered as the treatment of choice. However, nowadays, spleen-preserving management with percutaneous image-guided drainage is favored.^[36,37]^ Ultrasound-guided or CT-guided percutaneous drainage is advised for unilocular or bilocular abscesses^[22]^ that have a discrete wall and no internal septations with its liquid content being thin enough to be drained,^[38]^ whereas splenectomy for multiple abscesses is reported as a safe and effective treatment choice.^[29]^ However, our patient was not consenting with the use of ultrasound-guided pus drainage upon his first admission to our institution. The authors believe that the reason why the patient did not consent for the ultrasound-guided intervention is perhaps because of the lack of awareness of the general population about the new management modalities that interventional radiology offers. This lack of knowledge will decrease their probability of choosing interventional radiology as part of the initial management in this case.

Abscesses owing to *M tuberculosis* and *Candida*, medical treatment is more suitable.^[2,22,39]^ Failure of medical treatment will ultimately lead to surgical intervention.^[22]^ It is also important to note that in the chronic course of *Brucella* infections, abscesses should be managed with the combination of both medical and surgical treatment.^[22]^

Percutaneous drainage is more advantageous than surgical drainage as it spares the risks associated with a splenectomy. It is also cheaper and has a low risk of intra-abdominal spreading, the absence of postoperative complications, including those owing to anesthesia and wound infection, a shorter length of hospital stay, and better compliance of patients.^[4]^

The most common complications following percutaneous drainage are hemorrhage^[40]^ into adjacent organs, pneumothorax,^[40]^ pleural effusion, and colonic injury.^[41]^

Splenectomy is the treatment of choice for many surgeons. However, as in many cases of splenic abscesses, parasplenic inflammation together with extensive adhesions makes it challenging even to the experienced surgeon to perform a proper splenectomy. This is without forgetting that the mortality rate after surgery for splenic abscess varies from 13% to 30%,^[42,43]^ especially because of intra-abdominal perforation of the abscess or rupture of the spleen during the surgery. The suspicion of neoplasm arising from the splenic flexure of the colon invading the spleen was not considered as a possibility at the first surgery but rather, the splenic abscess caused an inflammation of the splenic flexure of the colon. Thus, cancer resection was not performed. A colonoscopy was recommended to him after the first intervention, and scheduled as appropriate but the patient did not attend that appointment. Unfortunately, the patient presented again within 48 days with liver metastases and during the second surgery, performed 115 days after the first intervention, the cancer was staged sT4NxM1 with a mass in the small intestines as well as liver metastasis and pelvic floor seedlings.

Successful splenectomy is also a case to warrant care as it entails the great risk of fulminant infections with incidence being 3.2% and mortality rate being 1.4%,^[44]^ with the most common pathogen being *Streptococcus pneumoniae, Haemophilius influenzae*, and *Neisseria meningitidis*. Most infections happen within 2 years post-splenectomy with also one-third of cases occurring up to 5 years post-splenectomy.^[45]^ Moreover, 38% to 69% of post-splenectomy patients who develop sepsis will die,^[44]^ despite the administration of pneumococcal vaccine and antimicrobial prophylaxis.

### 8.3 The involvement of the spleen with the carcinoma of the splenic flexure of the colon

In the case of an invading neoplasm, the formation of a splenocolic fistula is not impossible, which will lead to the formation of a splenic abscess with an air-fluid level as seen in our case. Including our case report, this will be the 13^th^ case report pertaining to invading colonic cancer causing a splenic abscess with respect to a PubMed search with key words “EXACT (splenic abscess) AND colonic cancer” accessed on July 30th, 2015. Relevant articles were those published in English and French or had abstracts in English or French, even though the articles were in any language other than French or English. This yielded the following relevant results, as shown in Table 3.^[46–57]^

**Table 3:**
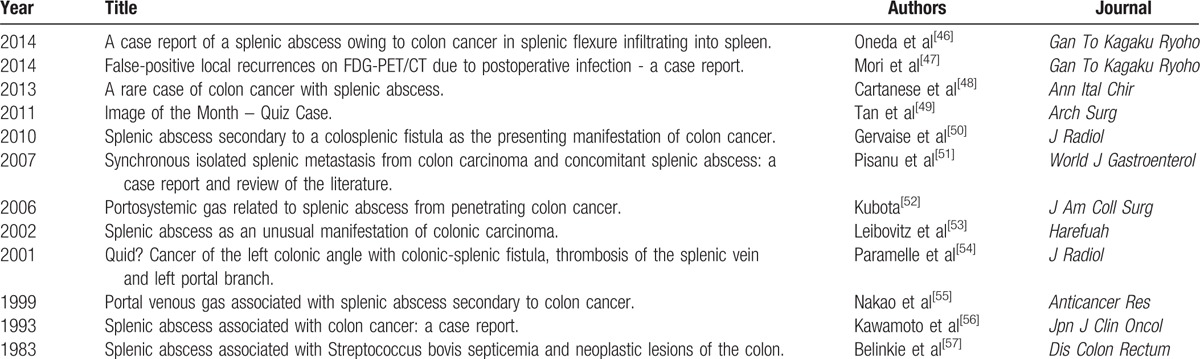
Relevant articles about carcinoma of the splenic flexure from a PubMed search with key words “EXACT (splenic abscess) AND colonic cancer” accessed on July 30th, 2015.

In 2014, Oneda et al^[46]^ reported a similar case as ours, with no metastases, which underwent a left hemicolectomy with splenectomy. Their histological diagnosis of mucinous adenocarcinoma was made with the pathological findings of pT4b, pN1, cM0, fStage IIIa. They^[46]^ recommended an en-block resection including lymph node resection.

Mucinous gastrointestinal tumors can cause perforation and infiltration into the full thickness of the bowel wall extending to the pericolic fat.^[58,59]^ Cabanas et al^[59]^ proposed that mucus-producing epithelial cells are entrapped within the trabeculae of the splenic capsule, in congenital clefts of the spleen and microfissures caused by trauma. From thereon, the mucinous tumor will expand into the soft splenic parenchyma rather than in the peritoneal cavity.^[59]^

Two particular case reports mentioned the presence of gas in the portal vein system with synchronous splenic abscess owing to local invasion of colon cancer,^[52,55]^ whereas there was a Polish article of splenic cancer complicated by perirenal abscess^[60]^ and 2 Spanish reports about intra-abdominal abscess from a nonspecified colon cancer lesion^[61]^ and 7 cases of cancer of the splenic flexure with 1 case re-operated for retroperitoneal abscess.

### 8.4 Metastasis to the spleen

Apart from the direct neoplastic invasion into the spleen, one should not forget the metastasis of cancerous cells to the spleen, although the spleen is an infrequent site despite its high vascularity. To attempt the explanation of the spleen being an infrequent site, there are various proposed hypotheses including the anatomical placement of structures, the rhythmic parenchymal sinusoidal contractions, and the absence of afferent lymphatic vessels in the parenchyma, among others.^[62–64]^

The spleen is the second largest organ of the reticuloendothelial system, which firstly blocks metastasis thanks to the anti-neoplastic properties of the lymphoid-rich splenic parenchyma. On an anatomical point of view, the sharp angle of the splenic artery with the celiac axis and the rhythmic contraction by the sinusoidal splenic architecture may limit the propagation of metastasis into the spleen.^[62,63]^ In addition, the splenic parenchyma contains no afferent lymphatic vessels, although they are present in the subcapsular, capsular, and trabecular regions. Yet, tumor cells can still reach the spleen through the lymphatic system, which explains the typical subcapsular isolated splenic metastases. Also, Indudhara et al,^[64]^ in 1997, proposed that neoplastic cells can reach the splenic vein and parenchyma by retrograde diffusion through the inferior mesenteric vein.

Splenic metastatic lesions can be solitary, multiple, or diffused and may vary in number and size. When present, it is not uncommon to find other metastatic sites such as lung, liver, and lymph nodes with common primary cancer sites being breast, lung, liver, stomach, pancreas, ovary, melanoma, and colon cancer.^[33]^ Splenic metastasis can be seen in 2% to 9% of untreated cancer patients but systemic chemotherapy has led to an increase in the incidence of splenic metastasis.^[65–67]^ Forty-one cases of splenic metastases with primary colon cancer were studied in a literature review by Pisanu et al^[51]^ in 2007, which included only 3 cases of synchronous colon cancer and splenic metastasis. Splenic abscess was also present in 2 cases, which had synchronous splenic metastasis with colon cancer in this review.

The incidence of such a complication does not occur on a daily basis and the ability of the clinician to correlate the different signs and symptoms together with relevant diagnostic tools comes with experience. With the increasing popularity in the Western world of interventional radiology, the Chinese population are still sceptic in accepting and understanding these new methods. However, given in our case, it is still debatable as to whether or not, depending on the clinical care setting, it was possible to employ the interventional radiology in contrast to the radical surgical approach. *Enterobacter cloacae* was the organism cultured from the pus drained intraoperatively from our patient. Coupled with the presence of the splenocolic fistula seen on CT scan, the presence of *Enterobacter cloacae*, which is part of the normal human gut flora in many humans,^[68]^ supports the pathological mechanism of direct invasion of the neoplastic splenic flexure into the spleen, into which the bacteria of the normal gut flora proliferated leading to abscess formation. The limitation of this case report was the missed appointment of the patient which could have resulted in, perhaps, more effective results to have been achieved. Furthermore, the diagnostic colonoscopy performed during his second admission did not point toward an overt splenocolic fistula, which again emphasizes the importance of user-operated imaging tools. This report is the 13^th^ of similar nature to increasing cases of similar nature with the hope to keep the physician aware of this rare, but yet tragic incident that can occur. The oncological timeline has not been included in this report as it, according to the authors’ perspective, goes beyond the essential diagnostic message that we wish to pass through.

## 9 Conclusion

The incidence of splenic flexure of the colon causing splenic abscess is a rare feat that has a growing number of reported literature over the years. With the advent of increasing immunosuppressed patients such as post-transplant, undergoing chemotherapy and AIDS, its incidence is increasing over the years. The splenic flexure of the colon still remains the least frequent site of occurrence of colonic neoplasm. Direct invasion into adjacent organs will cause atypical presentation of this condition, which will usually happen at an advanced stage of the disease. CT scan and ultrasound imaging modalities are pillars of diagnosis and are becoming the favorite approach for the initial step of treatment with image-guided percutaneous drainage over open surgery. The micro-organisms cultured in blood or pus samples in suspected splenic abscesses can hint toward the site of the primary insult. When surgery becomes the ultimate recourse for carcinoma of the splenic flexure, an en-block resection with splenectomy with or without lymph node resection, depending on the stage of the disease, is advised. However, there is not enough evidence to support or reject this approach because of the limited number of cases available in the literature. It is of utmost importance that any splenectomy should have the proper follow-up with respect to vaccination and warning against fulminant infections. The young and inexperienced surgeon must always keep in mind the probability, no matter how minimal, of the occurrence of a splenocolic fistula before it is too late. Again, emphasis cannot be put enough on the use of a multidisciplinary team for the proper detection and management of such cases.
